# Stable and luminescent halide perovskite fabricated in water

**DOI:** 10.1038/s41377-020-0340-x

**Published:** 2020-06-19

**Authors:** Guangren Na, Lijun Zhang

**Affiliations:** State Key Laboratory of Integrated Optoelectronics, Key Laboratory of Automobile Materials of MOE and College of Materials Science and Engineering, Jilin University, Changchun, 130012 China

**Keywords:** Optical materials and structures, Photonic devices

## Abstract

Lead bromide perovskite nanoparticles are fabricated in the water, which has been recognized previously as a severe source of damage to halide perovskite materials and devices. The perovskite nanoparticles exhibit a high photoluminescence quantum yield and excellent material stability.

The past decade has witnessed the dramatic development of hybrid lead halide perovskites as promising semiconductors for applications in high-performance solar cells, photodetectors, light-emitting diodes, etc^1^. While the optoelectronic conversion efficiency of this family of intensively investigated materials has been persistently improved, there are several challenges standing in the way of their practical application. Although lead-derived toxicity is still a concern^2–4^, poor stability is the most urgent problem confronting the halide perovskite research community^5,6^. The manifestation of the poor stability includes the visible degradation of materials and devices exposed to room and high temperatures, illumination, oxidizing environments, and humidity^7–10^, which is rooted in the not strong mixed ionic and covalent chemical bonding of halide perovskites.

To overcome the instability issue of halide perovskites, a few strategies have been developed, including A-site composition engineering, the addition of defect passivation or ion migration blocking layers, dimensional reduction to two-dimensional phases or nanoparticles, grain size engineering or fabrication of single-crystal materials^7–10^. Despite the progress to date, obtaining materials and devices with both an optimal optoelectronic performance and a long lifetime under working conditions remains challenging.

Among the factors that cause degradation of perovskite materials and devices, humidity has been recognized as a severe and irreversible source of damage. To achieve high-performance optoelectronic devices, the amount of water involved in the material synthesis and exposed to perovskite active layers in devices has to be strictly controlled. This is, however, not the case in a recent publication, where Liu et al. fabricated the lead bromide perovskite nanoparticles that show excellent stability and high luminescence performance by deliberately introducing a large amount of water^11^.

The fabricated methylamino lead bromide perovskite (MAPbBr_3_) samples are reaction products of ammonium ion modified MAPbBr_3_ and water, and are composed of MAPbBr_3_ nanoparticles encapsulated in PbBr(OH) matrix (Fig. 1). The PbBr(OH) encapsulation improves the material stability and luminescent behavior for several reasons: first, it acts as a blocking layer to prevent ions (e.g., MA^+^) from escaping to the air or migrating among grains; second, it can passivate nonradiative defects on the perovskite surface (e.g., bromine vacancy V_Br_) effectively; third, it will confine electron and hole states to enhance oscillator strength and intensity of optical transition; finally, it provides protection by isolating MAPbBr_3_ from sources of degradation, such as H_2_O/O_2_. Compared with the MAPbBr_3_ synthesized by the normal procedure, the fabricated samples exhibit a significantly enhanced photoluminescence quantum yield (PLQY) from about 2.5% to 71.54%. Meanwhile the PLQY of the samples decreases to only ~90% of its initial value after one-year storage in aqueous solution. The samples show good stability under humid, high-temperature, and illumination conditions.

**Fig. 1:**
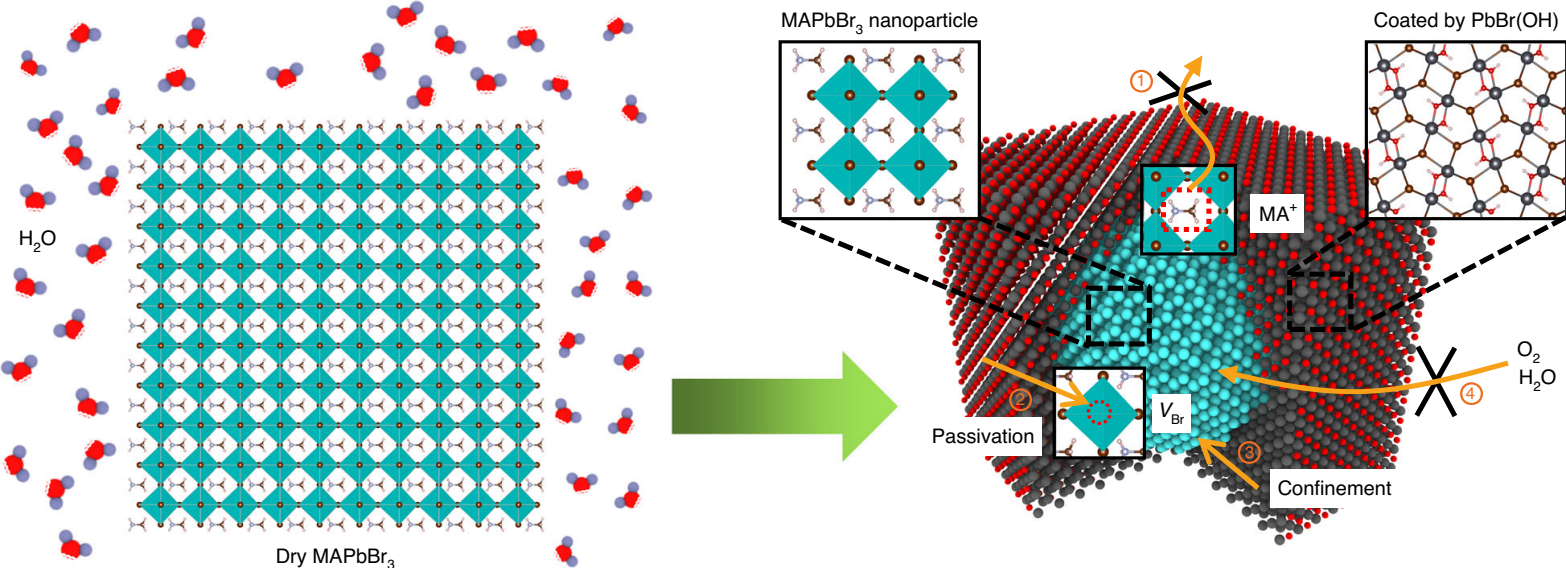
Schematic of the formation of MAPbBr_3_ perovskite nanoparticles encapsulated by PbBr(OH) layers from the reaction between ammonium ion modified MAPbBr_3_ and water. The four roles of the PbBr(OH) encapsulation layers in improving the material stability and luminescent behavior are indicated

The synthesis approach that involves water is universal to halide perovskites with different A-site cations. Moreover, it is facile and may be extended to large-scale production. This offers a new and feasible strategy to fabricate highly stable and luminescent halide perovskites and indicates that there are remaining options to be explored to improve the stability of this family of materials. Future research may be directed at optimizing the profile of PbBr(OH) encapsulation layers and the interface between them and halide perovskites to enable more efficient charge carrier injection, which may improve the performance of the materials and devices further.
